# Interventions to increase the distribution of vaccines in Sub-Saharan Africa: a scoping review

**DOI:** 10.11604/pamj.2019.32.14.17225

**Published:** 2019-01-09

**Authors:** Marius Zambou Vouking, Christelle Monique Angoula Mengue, Saidu Yauba, Jean Marie Edengue, Modibo Dicko, Hamadou Modibo Dicko, Charles Shey Wiysonge

**Affiliations:** 1 Center for the Development of Best Practices in Health, Yaoundé Central Hospital, Henri-Dunant Avenue, Messa, Yaoundé, Cameroon; 2 Central Technical Group of the Expanded Program on Immunization, Yaoundé, Cameroon; 3 University of Dschang, Department of Biomedical Sciences, Dschang, Cameroon; 4 Clinton Health Access Initiative, Country Office, Cameroon; 5 Ministry of Public Health, Yaoundé, Cameroon; 6 Health Supply & Solar Systems (H3S), Systèmes Solaires & Logistique de Santé, Cité El Farako, Bamako, Mali; 7 Global Alliance for Vaccines and Immunization (GAVI), Geneva, Switzerland; 8 Cochrane South Africa, South African Medical Research Council, Tygerberg, South Africa

**Keywords:** Increase, distribution, vaccines, Africa

## Abstract

Achieving universal access to immunization, as envisioned in the global vaccine action plan continues to be a challenge for many countries in Sub-Saharan Africa. Weak immunization supply chain (iSC) has widely been recognized as a key barrier, hindering progress towards vaccination targets in this region. These iSCs, which were designed in the 1980s, have become increasing fragile and are now considered outdated. The objective of this review was to assess the effectiveness of system redesign and outsourcing to improve outdated iSC systems in sub-Saharan Africa. We searched the following electronic databases from January 2007 to December 2017: Medline, EMBASE (Excerpta Medica Database), the Cochrane Library, Google Scholar, CINAHL (Cumulative Index to Nursing and Allied Health Literature), WHOLIS (World Health Organization Library Database), LILACS (Latin American and Caribbean Literature on Health Sciences) and contacted experts in the field. Our search strategy yielded 80 records and after assessment for eligibility, seven papers met the inclusion criteria. Five studies evaluated the experiences of system redesign in three countries (Nigeria, Benin and Mozambique), two assessed outsourcing vaccine logistics to the private sector in Nigeria and South Africa. According to these studies, system redesign improved vaccine availability at service delivery points and reduce the cost of distributing vaccines. Similarly, outsourcing vaccine logistics to the private sector reduced the cost of vaccines distribution and improve vaccine availability at service delivery points.

## Introduction

Vaccination coverage in Africa has improved dramatically since the Expanded Program on Immunization (EPI) was established in 1976. However, achieving universal access to immunization in this region, as envisioned in the global vaccine action plan (GVAP) continues to be a challenge for many countries [1]. In 2015, only 13% of countries in the region achieved the GVAP target of 80% coverage in every district or administrative equivalent with all EPI vaccines in their national programs, suggesting that the continent's progress toward global immunization goals is largely off-track [1]. Although several factors have been advanced for this failing, there is a growing recognition of the contribution of outdated immunization supply chain systems on missed opportunities for vaccination [2]. Indeed, these systems, which were designed in the late 1970s to manage fewer, less expensive and less bulky vaccines, are being confronted with several new realities [3]. Amongst these are the needs to handle a widening variety of new vaccines and immunization schedules, a greater diversity of service delivery strategies, an ever-expanding target population to vaccinate, and an increased cold chain infrastructure requirement [3]. Taken together, these factors have created significant storage and transport shortfalls at all levels, which ultimately hinder progress towards coverage and equity goals in the continent [4]. In 2016, only 19% of Gavi-supported countries in the region met with the 80% minimum threshold for the World Health Organization (WHO) recommendations for effective vaccine management (EVM) [2], representing a marginal improvement from 2010, where no country met the 80% bench mark across all nine EVM categories [5]. This marginal progress seems to indicate that adherence to many EVM policies, standards and quality management continues to be problematic in the region. Indeed, an earlier report has shown that over three in four African countries lacked adequate systems for proper vaccine handling, leading to recurrent stock outs, expired products, and stock damage during storage and transit, all of which contribute to missed opportunities for vaccination [2].

In 2014, for instance, 22% of African countries experienced district level stockouts, which interrupted immunization service delivery at facility level, leading to missed opportunities to vaccinate children [4]. Today, with few exceptions, immunization supply chains in this region faces chronic difficulties in providing uninterrupted availability of potent vaccines up to service delivery levels, and many government-managed systems remain crippled by inefficiencies in vaccine storage, distribution, vaccine management and stock control [3]. The global vaccine community has long recognized that, overcoming these challenges will require a radical shift in the way immunization supply chain systems are designed, managed, resourced and supported [2]. In 2013 a task force, involving four core Alliance partners, namely the Gavi secretariat, WHO, UNICEF and the Bill & Melinda Gates Foundation, was established to lead the development of an immunization supply chain strategy that leverages core capabilities and strengths of each organization to support and influence meaningful and measurable improvements in national immunization supply chains [6]. Key focus areas included developing strategies, which can improve vaccine adequacy and availability at the last mile. Many of these innovative strategies have been piloted in several countries, but little is known about their impact on various immunization outcomes. In this paper, we assessed the effectiveness of system redesign and outsourcing of vaccine logistics to the private to improve immunization outcomes in Sub-Saharan Africa.

## Methods

**Type of studies:** all study designs, which provided information on routine iSC and vaccine logistics in Sub Saharan Africa, were eligible for inclusion. This included randomized controlled trials, controlled/uncontrolled before and after trials, interrupted time series, cross-sectional studies, cohorts, and case control studies.

**Search strategy:** we searched the following electronic databases: Medline, EMBASE (Excerpta Medica Database), The Cochrane Library, Google Scholar, CINAHL (Cumulative Index to Nursing and Allied Health Literature), WHOLIS (WHO Library Database), LILACS (Latin American and Caribbean Literature on Health Sciences), GAVI, Technet21, BID Initiative, intraHealth, VillageReach and contacted two experts in the field. We used the following combination search terms: (“supply and distribution”[Mesh] OR materials management[tiab] OR (supply chain*[tiab] AND (redesign*[tiab] OR improv*[tiab) OR ((purchas*[tiab] OR procure*[tiab) AND (vaccines*[tiab) AND (“Cost Savings”[mesh] OR “Cost Benefit Analysis”[Mesh] OR “Eficiency”[Mesh] OR stock out*[tiab] OR stockout*[tiab] OR out of stock[tiab]). Our search period ran from January 2007 to December 2017.

**Data extraction and management:** the first author (MZV) screened initial results' titles and abstracts and excluded studies or reports that did not met our inclusion criteria (Table 1). Following this initial screening, two authors (MZV and CMAM) used a pre-defined template to independently extract data from studies that met our inclusion criteria. Extracted data included information on study design, study aims, location, population, intervention, and the impact on certain vaccination outcomes. Extracted data were independently checked by three authors (MZV, CMAM, JME) and the original reports were consulted by these authors for clarification if data were missing or unclear. Finally, we developed a data extraction form on Microsoft Excel as a systematic tool to collect the relevant data for our study (Table 2). Critical appraisal of all identified citations was done independently by two authors (MZV and CMAM) to establish the possible relevance of the articles for inclusion in the review. Disagreements were resolved by consensus or by arbitration of a third review author (JME). We retrieved full text copies of the articles identified as potentially relevant either by one or both review authors. Where appropriate, we contacted study authors for further information and clarification.

**Table 1:** Inclusion criteria

Peer-reviewed papers or report that were published between January 2007 and December 2017
Full text of peer-reviewed papers available in any language Peer-reviewed papers or report that specifically targeted one or several African countries
Peer-reviewed papers or report with a strong methodological background and/or providing useful finding directly related to immunization supply chain and logistics

**Table 2:** Description of studies

First Author – year	Title	Country of study	Type of intervention
Lee *et al.,* 2016	Re-designing the Mozambique vaccine supply chain to improve access to vaccines	Mozambique	Informed Push Model
Huang *et al.,*2017	Costing analysis and anthropological assessment of the vaccine supply chain system redesign in the Comé District (Benin)	Benin	Informed Push Model
Sarley *et al.,* 2017	Transforming vaccines supply chains in Nigeria	Nigeria	Informed Push Model and Outsourcing
Aina *et al.,* 2017	Preliminary results from direct-to-facility vaccine deliveries in Kano, Nigeria	Nigeria	Informed Push Model
Lee *et al.,* 2015	The Benin experience: how computational modeling can assist major vaccine policy changes in low- and middle-income countries	Benin	Informed Push Model
Wendy *et al.,*2017	System redesign of the immunization supply chain: Experiences from Benin and Mozambique	Benin and Mozambique	Informed Push Model
Lydon *et al.,*2015	Outsourcing vaccine logistics to the private sector: the evidence and lessons learned from the Western Cape Province in South-Africa	South Africa	Outsourcing vaccine logistics

## Current status of knowledge

**Figure 1:**
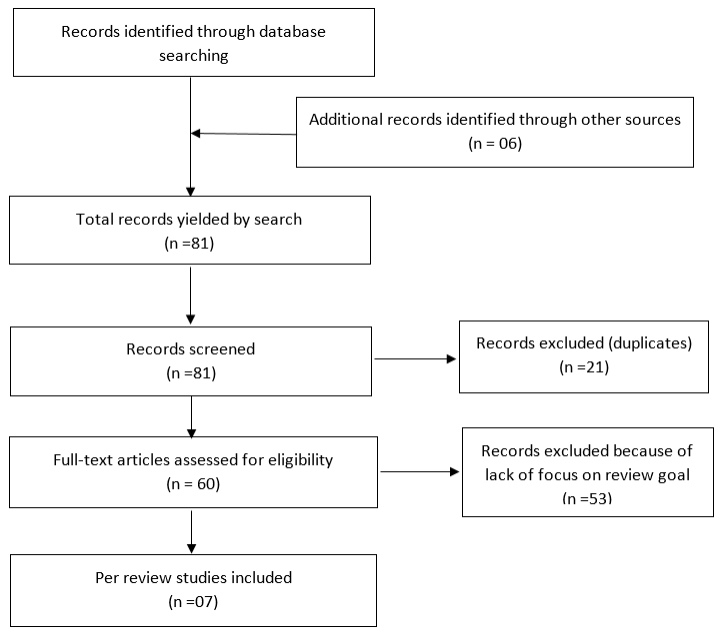
Prisma flow diagram

**Description of studies:** out of the 81 papers (of which, 21 duplicates were removed), we pre-selected 60 based on their title and abstract. After reading full text, we excluded 53 papers because their content did not match our review goals. As shown in Figure 1, just seven studies met our inclusion criteria, two of which were conducted in Benin [7, 8], two in Nigeria [9, 10], one in Mozambique [11], one in both Benin and Mozambique [12] and one in South Africa [13]. Furthermore, these studies were randomised trials [7-13]. One study assessed the cost of vaccine supply chain system redesign [8] while one study assessed outsourcing vaccine logistics to the private sector in South Africa [13].

**System redesign followed by informed push:** five studies evaluated the pilots of iSC system redesign in three African countries, namely Benin, Nigeria and Mozambique [7-11]. The Ministries of Health (MoH) in these countries undertook system redesign activities to address the longstanding underperforming iSC systems, which were originally designed with four tiers over four decades ago. In the intervention areas, the HERMES (Highly Extensible Resource for Modeling Supply Chains) simulation modeling tool was used to identify the most suitable design for improving the performance of iSC in terms of vaccine adequacy and availability, logistics cost per dose administered and immunization coverage. In Benin, the modeling results suggested that the most efficient system redesign would require consolidating sub-district vaccine stores to one district vaccine store and introducing truck loops [7]. In addition, the results identified an informed push design to be the most efficient and feasible form of vaccines distribution. The model was approved by MoH and piloted in 2013, in Comé District in the Mono-Couffo division. In the pilot, high performing cold chain equipment were first installed to address storage gaps. This was immediately followed by the introduction of an informed push distribution model, which enabled vaccines to be directly delivered from Comé district to 37 health facilities using real-time data from those facilities. Deliveries were grouped into transit trajectories (stores per health zone), according to an efficiently planned itinerary, so that each trajectory serves a maximum number of health facilities depending on the storage capacity of the refrigerated vehicle dedicated for vaccines delivery. A number of supply chain managers were trained and tasked with collecting vaccines from the regional store, conducting monthly visits to each health facility for vaccine distribution, retrieve used safety box and data collection, providing supportive supervision and analyzing data for improved distribution planning. Vaccinators from eight facilities also received refresher trainings on EVM.

Like in Benin, HERMES software was used to propose a re-design model of the Mozambique iSC, which was then piloted in Gaza and Cabo Delgado provinces [12]. In the Capo Delgado province, the re-design included three distribution loops, delivering vaccines monthly from the provincial level directly to 111 (52%) health centres in the province. In Gaza, the redesign was adapted to the difficult terrain, taking into account small populations dispersed over long distances. In this region, the primary redesign utilized two distribution loops to deliver vaccines from the provincial level directly to health centres in the southern region while two additional loops delivered vaccines to facilities in the northern region. In both provinces, the alternative design eliminated the district level as a distribution point, which became a warehouse for emergency stock only. Logisticians were trained on key aspects of the new system and tasked with key responsibilities, which included direct distribution to health facilities, direct data collection from health facilities, and mentoring of facility staff. New cold chain equipment were also installed where needed. In Nigeria's Kano State, the re-designed eliminated 44 Local Government Authority cold stores, enabling vaccines to be pushed directly from 6 state stores to primary health centres [9]. First, target centres were equipped with solar refrigerators in line with the national policy of having one fully equipped and functional solar refrigerator per political ward of primary health centre. Secondly, trained officers conducted bi-weekly deliveries directly from the state store to these facilities before transitioning to monthly deliveries. This transition was necessary so as to decrease overall program costs.

**Outsourcing vaccine logistics to the private sector:** two studies assessed the outsourcing of vaccine logistics to the private sector in Nigeria and South Africa [10, 13]. In Nigeria, Aina *et al.* documented the experiences of redesigning the supply chain system prior to outsourcing vaccine distribution to the private sector [10]. In this pilot, stock holding levels at some local government areas in Kano state were eliminated and an informed push model, known as “PUSH Plus” was recommended for testing in public health facilities in three government areas. Vaccine distribution to these facilities was outsourced to a private transporter, Tranex, who in turn conducted bi-weekly deliveries of vaccines and vaccination consumables directly from the state cold store to the target facilities [10]. In 2015, Lydon *et al* assessed the experiences of Western Cape Province that outsourced certain supply chain functions to a third-party logistics service provider [13]. This decision was driven by the need to overcome perennial challenges of stock management, inadequate cold chain storage at facilities, and overall inefficiencies in the government run distribution system for vaccines [13]. The private sector logistic provider, which had a comparative advantage in storing, handling and transporting vaccines at lower costs and at higher levels of service, was responsible for providing the necessary cold storage space and managing vaccines for the province, including ensuring effective vaccine transport from the provincial warehouse directly to health centres [13].

### Effects of interventions

**System redesigned followed by Informed Push Model:** the effects of system redesign was measured differently in different settings. In Benin, outcomes were assessed by comparing scores from EVM assessment conducted at baseline and endline [12]. Overall, EVM scores significantly improved between baseline and endline across all 9 criteria in Come district but improvements were more marked with focus criteria of the pilot [12]. Indeed, EVM scores for distribution rose from 40% to 100%, vaccine management practices increased from 58% to 98% and infrastructure from 55% to 94% [7]. Furthermore, the scores observed in Come district were significantly higher than those in the control district in all criteria, with unmatched differences in distribution (100% versus 32%), vaccine management (94% versus 63%) and maintenance (79% versus 6%). In addition, improved EVM scores were observed at the health facility level, and scores exceeded the recommended 80% for five of the eight criteria as opposed by none in the baseline study. Finally, facilities in Comé district significantly outperformed the control districts in all EVM criteria [12]. In Mozambique, outcomes other than EVM were used to measure the impact of the iSC redesign [12]. In the pilot province, DTP-3 coverage in children aged 12-23 months increased by nearly 24 points, rising from 68.9% at baseline to 92.8% at endline (OR 5.8, 95% CI 3.2-10.5) [12]. Drop-out rates between DTP-1 and DTP-3 plummeted by 8.2 points, falling from 12% at baseline to 3.8% at endline. Furthermore, stockouts rates reduced from 79% at baseline to less than 1% at endline [12]. Over 96% of intervention facilities had a functional refrigerator one year after the pilot had ended. Overall, the improvements observed in the pilot provinces were superior to those in the control provinces [12].

In Nigeria, stock adequacy and stock-outs were the primary outcome measured during the pilot [10]. Overall, stock adequacy improved by 14 points, rising from 54% at baseline to 68% at endline [10]. In contrast, stock-out rates plummeted by 31 points, falling from 41% at baseline to 10% at endline [10]. Similar trends were observed in analysis restricted only to facilities that had cold chain equipment at the start of the intervention, with stock-outs decreasing from 40% to 13%, and stock adequacy increasing from 46% to 70% over the period [10]. In addition to the above improvements, the reviewed studies-including an anthropological study-unveiled other important benefits of redesigning outdate and failing iSC [8]. In pilot facilities, a dramatic improvement in the frequency of immunization services was observed across all sides [8]. This improvement was attributed to improved vaccine availability and staff time to care for patients. The latter has been linked to the relieve of facility staff from the burden of vaccine collection as well as the elimination of other risks that staff faced during vaccine collection-notably the risk of injuries from road traffic accidents [8]. Improvements in staff motivation and professional awareness emanating from trainings, supportive supervision, and improved work conditions were also noted [8].

**Outsourcing vaccine logistics to the private sector:** scores from EVM assessments were used to measure the outcome of the outsourcing arrangement in South Africa [13]. According to the authors, the overall EVM scores for the outsourced iSC segments covered exceeded the 80% as opposed to 63% in the segment managed “in-house” [13]. Although, the private provider outperformed the government run supply chain in 6 EVM dimension, the difference was mostly marked on the criteria for temperature monitoring, vaccine distribution, and vaccine logistics information systems. However, it is worth indicating that the government-run segment outperformed the private sector in two EVM dimensions-sufficient cold chain storage capacity and compliance with vaccine management policies [13]. In Nigeria, stock-out rates and immunization coverage were used to measure impact of outsourcing in pilot health facilities [9]. In the pilot areas, the percentage of facilities with vaccine stock-outs dropped from 43% to 0%. At the same time, immunization coverage in these facilities rose from 57% to 88% [9].

**Cost of implementing the interventions:** financial data were collected and analysed differently by different authors. This limitation makes direct comparison of the cost of implementing iSC interventions challenging across the different pilots. Despite this limitation, the overall trend suggested cost savings for the governments. In Mozambique, cost per child vaccinated with DPT-3 was US$5.03 in the pilot province compared to US$6.07 in the control province, suggesting that iSC redesign was 17% more cost-effective than the program [12]. In addition, the cost per dose delivered was US$1.18 in the pilot province compared to US$1.50 in the control province, indicating that the pilot was 21% less expensive [12]. In Benin, the cost per dose administered decreased from US$0.14 at baseline to US$0.13 after eliminating the regional ISC tier. In Nigeria, the overall weighted average cost per delivery was US$29.8 [12]. The cost of outsourcing the vaccine delivery services following system redesign was initially US$15.1 (N5,462) per facility per month but this reduced by 15% to US$12.7 (N4,600) per facility per month as more health facilities were added [10]. The cost-efficiencies found in these pilots suggest that system redesign can improve supply chain performance while reducing overall logistic costs. However, it is noteworthy these may not reflect precise cost incurred in the pilots as several variables (e.g. variables in local salaries, operating cost and cost of goods) appeared not to have been included in the analysis [10]. This limitation highlights the need for further research into this area.

**Strengths and limits of the scoping review:** this paper represents, to our knowledge, the first attempt to review interventions that have been piloted, with the aim of improving the performance of iSC in Africa. The findings of the review may be relevant to scholars and policymakers, who have a strong interest in improving immunization coverage. Despite this strength, the paper has some important limitation. First, only 7 reports were included in this review. This limited sample size implies that the findings in this review should be viewed with caution because the pilots were conducted in settings with different realities, in terms of size, terrain, population, socio-economic development and health systems. Furthermore, we chose to limit the investigation to the geographic area of Africa, implying that we may be lacking the experience of iSC interventions in other continents.

**Implications for practice:** despite the small sample size, our review provides preliminary evidence from multiple settings that system redesign and outsourcing distribution can improve vaccine availability at the last mile and thus immunization coverage in Africa at a reduced cost. A noteworthy observation from Benin and Mozambique is that increasing the number cold chain equipment might not significantly improve vaccine availability in areas where distribution systems are weak. This is particularly important in the context of the Gavi-funded cold chain equipment optimization platform (CCEOP) project, which seeks to provide facilities with optimal equipment that are appropriate for their operational context. Building from the experiences in our review countries, we strongly recommend that system redesign should be considered as part of the package for CCEOP implementation. In addition, we recommend the conduct of well-designed control trials in different settings to further evaluate the impact of system redesign and outsourcing in Africa as evidence from these trials may support the development, roll out and monitoring and evaluation of evidence-based national iSC policies. For ease of comparison, we recommend researchers to use the EVM tool to evaluate the impact of the interventions, including their strengths and weaknesses as well as the ease with which they can be integrated into existing logistic management information systems.

## Conclusion

Although there is little published evidence on successful interventions to increase vaccines distribution in sub Saharan Africa, our review has identified success stories in few countries in the continent. Pilots in these countries suggest that system redesign and outsourcing vaccine logistics to a third party can improve vaccine availability, decrease stock outs and contribute to improvements in vaccination coverage. These interventions were also associated with other indirect benefits such as improved staff motivation and professional awareness, emanating from trainings, supportive supervision, and improved working conditions. Furthermore, the cost-efficiencies found in these pilots suggest that these interventions can improve supply chain performance while reducing overall logistic costs. Despite these promises, there are apparently clear knowledge gaps, highlighting the need for more research in this important area that has been undermining progress towards universal vaccine coverage and equity goals.

### What is known about this topic

- Interventions to improve the availability of vaccines are commonly grouped into those targeting health services for delivery or supply;
- The most recent review considered showed that much has to be done to improve availability of vaccines in Sub-Saharan Africa.

### What this study adds

- There is insuficient evidence to support or refute the use of informed push model approach in improving vaccine availability at delivery points in Sub-Saharan Africa;
- There is insuficient evidence to support or refute the use of outsourcing vaccine logistics to the private sector in Sub-Saharan Africa.

## Competing interests

The authors declare no competing interests.
